# Alcoholic-Hepatitis, Links to Brain and Microbiome: Mechanisms, Clinical and Experimental Research

**DOI:** 10.3390/biomedicines8030063

**Published:** 2020-03-18

**Authors:** Manuela G. Neuman, Helmut Karl Seitz, Samuel W. French, Stephen Malnick, Heidekazu Tsukamoto, Lawrence B. Cohen, Paula Hoffman, Boris Tabakoff, Michael Fasullo, Laura E. Nagy, Pamela L. Tuma, Bernd Schnabl, Sebastian Mueller, Jennifer L. Groebner, French A. Barbara, Jia Yue, Afifiyan Nikko, Mendoza Alejandro, Tillman Brittany, Vitocruz Edward, Kylie Harrall, Laura Saba, Opris Mihai

**Affiliations:** 1 In Vitro Drug Safety and Biotechnology, Toronto, ON M5G 1L5, Canada; m_neuman@rogers.com; 2 Department of Pharmacology and Toxicology, Faculty of Medicine, University of Toronto, Toronto, ON M5G 1L5, Canada; 3 Department of Medicine, Centre of Alcohol Research, University of Heidelberg, Salem Medical Centre, 337374 Heidelberg, Germany; helmut_karl.seitz@urz.uni-heidelberg.de (H.K.S.); sebastian.mueller@urz.uni-heidelberg.de (S.M.); 4 Department of Pathology, Harbor-UCLA Medical Center and Los Angeles BioMedical Institute, Torrance, CA Harbor-UCLA Medical Center, Torrance, CA 90509, USA; sfrench@lundquist.org (S.W.F.); bfrench@lundquist.org (F.A.B.); yjia@dhs.lacounty.gov (J.Y.); nikoo.afifiyan@lundquist.org (A.N.); asmendoza@yahoo.com (M.A.); btillman@lundquist.org (T.B.); evitocruz@dhs.lacounty.gov (V.E.); 5 Department Internal Medicine C, Kaplan Medical Centre and Hebrew University of Jerusalem, Rehovot 76100, Israel; stevethemal@icloud.com; 6 Southern California Research Center for ALPD and Cirrhosis, Department of Pathology, Keck School of Medicine of the University of Southern California, Los Angeles, CA 90089-5311, USA; htsukamo@med.usc.edu; 7 Department of Veterans; Affairs Greater Los Angeles Healthcare System, Los Angeles, CA 90073, USA; 8 Division of Gastroenterology, Sunnybrook Health Sciences Centre, Department of Medicine, Faculty of Medicine, University of Toronto, Toronto, ON M4N 3M5, Canada; lawrence.cohen@sunnybrook.ca; 9 Department of Pharmacology, University of Colorado School of Medicine, Aurora, CO 80045-0511, USA; paula.hoffman@ucdenver.edu (P.H.); boris.tabakoff@ucdenver.edu (B.T.); kylie.harrall@ucdenver.edu (K.H.); laura.saba@ucdenver.edu (L.S.); 10 College of Nanoscale Science and Engineering, SUNY Polytechnic Institute, Albany, NY 12205, USA; mfasullo@sunypoly.edu; 11 Departments of Pathobiology and Gastroenterology, Center for Liver Disease Research, Cleveland Clinic Foundation, Cleveland, OH 44195, USA; len2@po.cwru.edu; 12 Department of Biology, The Catholic University of America, Washington, DC 20064, USA; tuma@cua.edu (P.L.T.); jenn.groebner@nih.gov (J.L.G.); 13 Department of Medicine, University of California, San Diego, La Jolla, CA 92093, USA; beschnabl@ucsd.edu; 14 Department Family Medicine Clinic CAR, 010164 Bucharest, Romania

**Keywords:** alcoholic hepatitis, acetaldehyde dehydrogenase (ALDH), alcohol dehydrogenase (ADH), CYP 1A1, CYP 1A2, CYP2E1, hepato-carcinogenesis, hepatocytotoxicity, his3-Δ3′ and his3*-* Δ5′, microsomal ethanol oxidizing system (MEOS), immunohistochemistry, laboratory markers, mithocondrion

## Abstract

The following review article presents clinical and experimental features of alcohol-induced liver disease (ALD). Basic aspects of alcohol metabolism leading to the development of liver hepatotoxicity are discussed. ALD includes fatty liver, acute alcoholic hepatitis with or without liver failure, alcoholic steatohepatitis (ASH) leading to fibrosis and cirrhosis, and hepatocellular cancer (HCC). ALD is fully attributable to alcohol consumption. However, only 10–20% of heavy drinkers (persons consuming more than 40 g of ethanol/day) develop clinical ALD. Moreover, there is a link between behaviour and environmental factors that determine the amount of alcohol misuse and their liver disease. The range of clinical presentation varies from reversible alcoholic hepatic steatosis to cirrhosis, hepatic failure, and hepatocellular carcinoma. We aimed to (1) describe the clinico-pathology of ALD, (2) examine the role of immune responses in the development of alcoholic hepatitis (ASH), (3) propose diagnostic markers of ASH, (4) analyze the experimental models of ALD, (5) study the role of alcohol in changing the microbiota, and (6) articulate how findings in the liver and/or intestine influence the brain (and/or vice versa) on ASH; (7) identify pathways in alcohol-induced organ damage and (8) to target new innovative experimental concepts modeling the experimental approaches. The present review includes evidence recognizing the key toxic role of alcohol in ALD severity. Cytochrome p450 CYP2E1 activation may change the severity of ASH. The microbiota is a key element in immune responses, being an inducer of proinflammatory T helper 17 cells and regulatory T cells in the intestine. Alcohol consumption changes the intestinal microbiota and influences liver steatosis and liver inflammation. Knowing how to exploit the microbiome to modulate the immune system might lead to a new form of personalized medicine in ALF and ASH.

## 1. Introduction

For the last 12 years, scientists from around the world have been participating in symposia dedicated to the scientific and clinical research of Charles S. Lieber. The focus is on the unwanted effect of alcohol on organ systems. Lieber’s contemporaries, as well as new researchers in the field, take the opportunity to present their innovative work in this forum. 

Alcohol (alcohol ethylic, ethanol) misuse may be considered as the most important hepatotoxin to which man has been exposed to since ancient times [1]. Alcohol-induced liver disease (ALD) consists of alcoholic fatty liver (AFL), acute alcoholic hepatitis (AH) with or without liver failure, alcoholic steatohepatitis (ASH) leading to fibrosis and cirrhosis, and hepatocellular carcinoma (HCC). Interestingly 10–20% of heavy drinkers (persons consuming more than 40 g of ethanol/day) may develop liver disease. Alcohol is the major etiologic factor of cirrhosis, which represents one of the most important causes of death.

The present review includes evidence recognizing the key toxic role of alcohol in ALD severity. Cytochrome p450 (CYP) 2E1 activation may change the severity of ASH and NASH. The microbiota is a key element in immune responses, being an inducer of proinflammatory T helper 17 cells and regulatory T cells in the intestine. The hepatotoxic effects on the liver structure of parenchymal cells (hepatocytes) as well as on non-parenchymal cells such as macrophages (Kupffer cells), hepatic stellate cells (Ito cells), and liver sinusoidal endothelial cells lead to architectural changes in the organ. Moreover, alcohol consumption changes the intestinal microbiota, and the toxic products of the microbiome (endotoxin) influence liver inflammation.

We aimed to (1) describe the clinico-pathology of ALD, (2) examine the role of immune responses in the development of alcoholic hepatitis (ASH), (3) propose diagnostic markers of ASH, (4) analyze the experimental models of ALD, (5) study the role of alcohol in changing the microbiota, and (6) articulate how findings in the liver and/or intestine influence the brain (and/or vice versa) on ASH; (7) identify pathways in alcohol-induced organ damage, and (8) target new innovative experimental concepts modeling the experimental approaches. The present review includes evidence recognizing the key toxic role of alcohol in ALD severity. Cytochrome p450 CYP2E1 activation may change the severity of ASH. 

## 2. Charles Lieber’s Scientific Legacy

Ethanol is metabolized in the liver to acetaldehyde [2]. This pathway is catalyzed in the cytosol by the alcohol dehydrogenase (ADH). The acetaldehyde is metabolized to acetate in the mitochondria by acetaldehyde dehydrogenase (ALDH). Both ADH and ALDH activities differ in genetically-diverse populations [2,3]. In 1968, C.S. Lieber and L.M. DeCarli published the classical discovery showing that the liver microsomes can oxidize ethanol (EtOH). They named it the “microsomal ethanol-oxidizing system (MEOS)”; the concept was very much discussed in that period of time. However, the discovery of the cytochrome p450 (CYP) 2E1-dependent MEOS explains ultrastructural, pharmacological, and biochemical effects of ethanol [3,4,5,6].

Acetaldehyde is generated via ADH and MEOS. Chronic alcohol misuse leads to MEOS induction that accelerates the metabolism of ethanol and facilitates organ injury. Cytochrome P450 CYP 2E1 induction leads to the formation of reactive oxygen species (ROS).

ROS includes radicals such as the ethoxy radical, hydroxyethyl radical, acetyl radical, superoxide radical, hydrogen peroxide, hydroxyl radical, alkoxyl radical, and peroxyl radical [7]. These radicals damage liver cells directly or via immuno-mediators such as cytokines and chemokines [8]. In susceptible individuals, alcohol misuse leads to liver injury, including fibrosis and cirrhosis [9].

Through CYP 2E1-dependent ROS, alcohol generates lipid peroxides and modifies the intestinal microbiome, thereby stimulating actions of endotoxins produced by intestinal bacteria. Endotoxins can be measured in the blood of alcoholics and constitute potential causes of liver injury [10,11].

Nutrition has an important role in the development of severe liver injury [12,13,14]. Lieber’s team experimentally demonstrated that replacement of dietary long-chain triglycerides (LCT) by medium-chain triglycerides (MCT) could be protective in ASH [15].

In mitochondria, 4-hydroxynonenal (4-HNE), a parameter of oxidative stress, parallels CYP2E1 activation. As a result, mitochondria contribute to lipid peroxidation and glutathione GSH depletion [14,15,16,17].

Chronic alcohol consumption increases the severity of viral hepatitis B (HBV) and C (HCV), in addition to co-infection of HBV or HCV with human immunodeficiency virus (HIV) -induced injury and contributes to adverse events in individuals taking anti-retroviral medication [18,19,20,21]. 

Monitoring liver stiffness is important for understanding disease severity. Introduced in 2003, Vibration-Controlled Transient Elastography (Fibroscan^®^, Echosens, France) technology is now widely used in routine clinical practice and research. A large number of studies demonstrated that liver stiffness measurement significantly correlates with liver fibrosis [22].

Treatment of ALD requires a multidisciplinary team involved in managing alcohol use disorder (AUD) as well as nutritional, toxicological, pharmacological, clinical and surgical intervention, as described and proposed by the guidelines of the American and the European Associations for the Study of Liver Diseases (AASLD, EASL) [23].

ALD is one of the major lethal outcomes of alcohol use, and the burden of disease is mainly due to (premature) years of life lost [24]. The global burden of the ALD is increasing [24]. In Denmark, from 1999 to 2011, although the consumption of alcohol did not change, the annual incidence rate of AH increased to 24 to 34 per million for men and women, respectively [25]. Moreover, among heavy drinkers, patients with AH have the fastest progression to end-stage liver disease [26]. In Metropolitan France, a study of 26,356,361 individuals (all acute inpatients and rehabilitation care patients) followed (2008 to 2012) showed that women with alcohol use disorders had 13 to 20-fold elevated risks for liver disease [26]. Also, the risk of death was age-related. In 20year-old women, the risk was 2.15%, while at the age of 53, the rock was 4.92%. The risk increase for the same ages was 1.98 to 3.97% in men. In the US there were 56,809 hospital patients for alcoholic hepatitis, with in-hospital mortality at 6.8% [27].

## 3. Interleukin-8 Signaling Pathway in Alcoholic Hepatitis 

Interleukin 8 (IL8) - CXCL8 is a potent chemotactic factor for neutrophils [28]. IL-8 binds to the chemokine receptors CXCR1 and 2 [3,29,30]. The CXCR1/CXCR2 receptors mediate the CXCL8-induced chemotaxis [31]. Also, LIMK2 is recruiting neutrophils to form an inflammatory infiltration [32]. The role of neutrophils in liver diseases [33], and in alcoholic hepatitis (AH) in particular, have been reviewed [34,35,36].

In a mouse model of alcoholic hepatitis (AH), [37] pepducin’s blockade of the CXCL-8 receptors 1 and 2 prevented the development of AH. The neutrophilic infiltrate was also reversed in AH after pepducin therapeutic intervention.

The two receptors CXCR1/2 are critical for the recruitment and activation of neutrophils. Pepducin therapy also decreased the transcription of liver CXCL-8 as well as abrogated transcription of IL-1B/CXCL-1 and TNFα and down-regulated caspase 1 expression.

Hep3b and HepG2 cells secreted CXCL-8 and attracted neutrophils, via chemotaxis, in response to ethanol exposure [37].

In liver biopsies from AH individuals, RNA sequencing was performed to study the changes in the expression of the IL-8 signaling pathway [38]. Induction of the IL-8 and CXCR2 in the livers of AH and controls as well as DDC re-fed mice and controls analysis employed quantification of mRNA carried out by SYBR real-time PCR assay. CXCR2 expression increased x 6 compared to control biopsies (*p* < 0.001). IL-8 expression was up-regulated 12 fold. The results showed that IL-8 was responsible for the neutrophil infiltration observed by histology as previously reported [39]. In addition, the genes in the IL-8 signaling pathway were up-regulated inducing LIMK2 (x 17.5), (canine nucleotide-binding protein Alpha 15) (GNA15) (x 27.7)), PIK3CB (x 8.5) and GNG2 (x 49).

**Figure 1:**
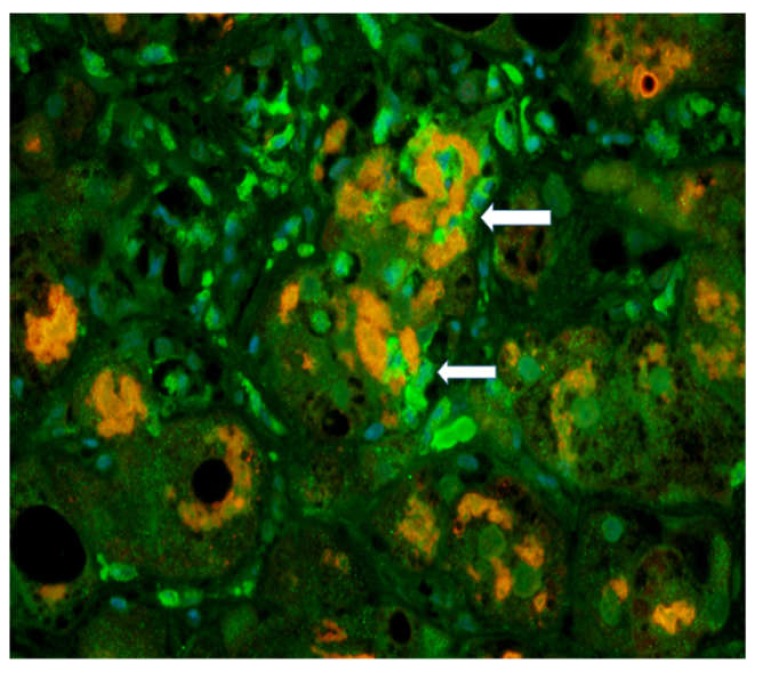
Immunofluorescent (IHC) staining of polymorpho-neutrophil cells (PMN)s with a GNA15 antibody showing neutrophils (arrows) (green) and Mallory Denk Bodies (orange stain) showing satellitosis × 200. Original figure is adapted from (Luo C, et al. 2018. Exp Cell Res 365: 1-11). No copywritting permission is required.

The antibody for GNA15 (canine nucleotide-binding protein Alpha 15) was used to stain the neutrophils in the liver biopsies. The antibody to ubiquitin was used to stain MDBs using a double IHC stain (Figure 1).

MDB forming hepatocytes were surrounded by neutrophils. The expression of GAN15 was up-regulated in AH biopsies compared to control livers.

**Figure 2:**
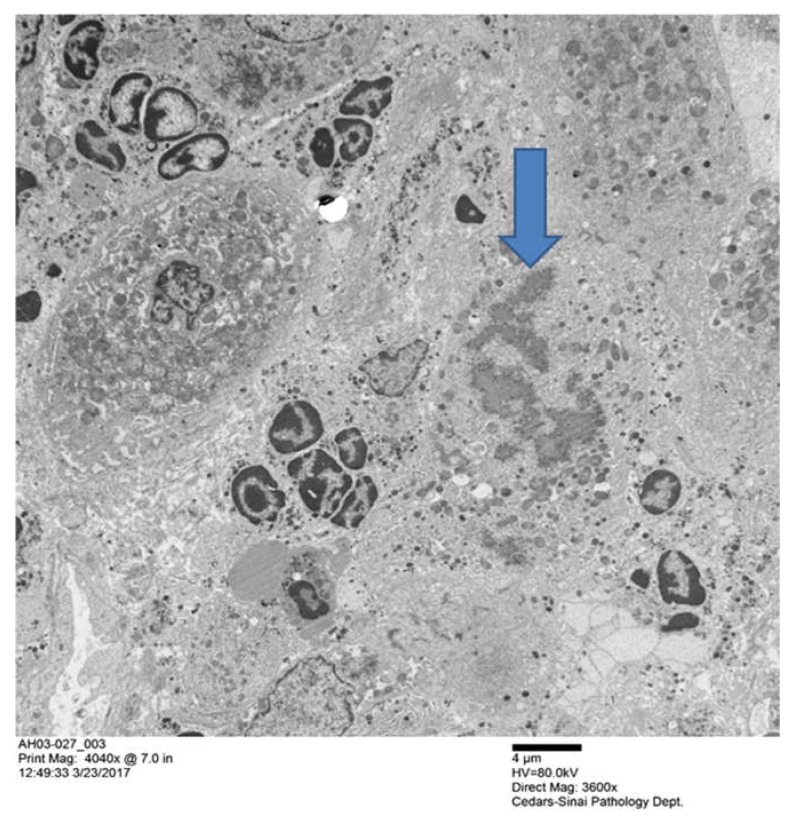
Groups of polymorpho neutrophils (PMN)s with condensed chromatin nuclei, clustered around two hepatocytes that had formed MDBs. The arrow pointed the MDB the MDB (× 4040).

The biopsy slide was stained with an antibody to myeloperoxidase as well as Hematoxylin & Eosin (H&E) staining [39] showing numerous neutrophyils, similar to those seen in Figure 1. This confirms that the GNA15 stained positive for neutrophyils. The myeloperoxidase stain also showed that sinusoids were filled with numerous neutrophyils. Electron microscopy of the liver biopsies showed satellitosis of the granulocytes around and next to the MDB-forming hepatocytes (Figure 2).

IL-8 was present in the cytoplasm of liver biopsies containing MDBs, indicating that IL-8 is involved in the pathogenesis of the neutrophylic infiltrate and MDB formation [39]. Modulation of the IL-8 pathway in AH livers with MDBs changes the IL-8 signaling pathways compared to healthy control livers. Several pathways downstream of IL-8, such as LIMK2 (chemotaxis), GNG2 (angiogenesis), and PIK3CB (survival, proliferation and mobility), lead to the activation of leukocyte recruitment [39].

## 4. FAT 10 in Alcoholic Hepatitis Pathogenesis

**Figure 3:**
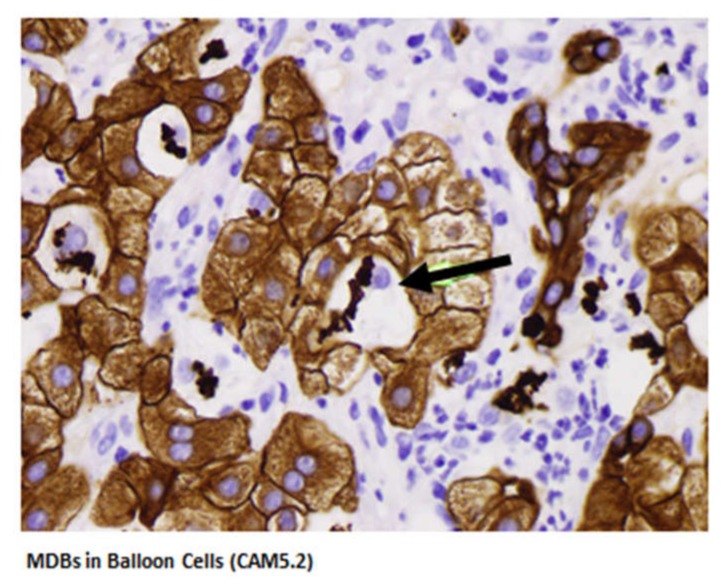
MDBs in balloon cells (CAM5.2). The black arrow points to the balloon cells with MDBs. The green arrow points to the bile duct metaplasia seen in alcoholic hepatitis. The lymphocytes (CD4) are nibbling the hepatocytes at the MHC antigen binding sites (immunologic synapsis) to gradually remove the hepatocytes (× 640).

The human leukocyte antigen F locus adjacent transcript 10 (FAT 10) is the only ubiquitin-like modifier which directly targets its substrate proteins for rapid degradation by the proteasome. FAT10 expression was measured in clinical trial-derived liver biopsies from patients with clinical evidence of alcoholic hepatitis (Table 1). Morphologic features of AH balloon cells forming Mallory-Denk bodies and bile duct metaplasia are shown in Figure 3. These two morphologic features are consistently found in AH.

**Figure 4:**
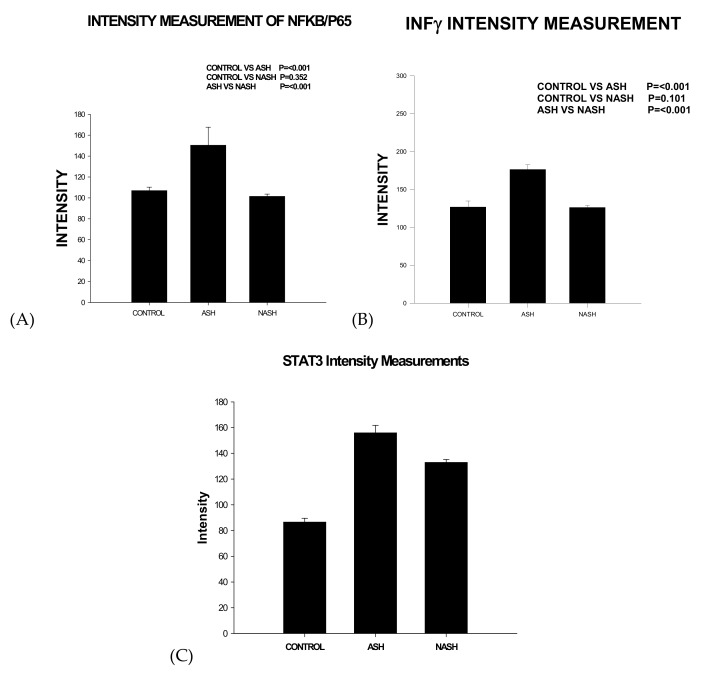
FAT10 expression is up-regulated by IFNγ and TNFα in intissue and express by intensity Intensity measurements in control, ASH, and NASH: (**A**) NFKb; (**B**) IFN γ; (**C**) STAT3.

FAT10, when expressed in high levels, has been shown to result in increased mitotic non-disjunction and chromosome instability leading to tumor genesis [40]. FAT10 expression is up-regulated by IFNγ and TNFα in all cell types and tissues (Figure 4). FAT10 is an oncogene that is associated with cellular malignancy, probably through its interaction with mitotic arrest-deficient 2 (MAD2) MAD2, p53, or β-catenin are under the influence of FAT10. This interaction leads to enhanced proliferation, invasion, and metastasis of cancer, as well as non-malignant cells [41]. FAT10 is associated with cellular malignancy, probably through its interaction with MAD2 [42]. However, other FAT10 pathways also lead to tumorigenesis, such as NFκB, Wnt signaling, and SMAD2 [41]. FAT10 up-regulation is critical for GRP78-mediated HCC proliferation. GRP78 modulates FAT10 expression via the NFκB signal pathway [43]. The FAT10 gene expression is up-regulated in 90% of hepatocellular carcinomas [44]. 

In the case of AH, FAT10 is essential to maintain the function of liver cell protein quality control and Mallory-Denk body (MDB) formation. This is based on KOFAT10 mice, which fail to form MDBs when fed DDC for 10 weeks [45]. The mechanism that is responsible for the loss of the ability to form MDBs is found in the FAT10 promoter of humans and mice, where the interferon sequence response element (ISRE) is located [46].

Several genes including NFκB, are expressed in response to the up-regulation by TNFα and IFNγ. FAT10 also signals a switch in the 26s proteasome’s 3 proteases to replace them with the 3 immunoproteases (MEK-1, LMP2, LMP7) [46]. The replacement of proteosome with immuno-proteases causes a failure to turnover proteins, leading to their accumulation and stabilization [45,46,47]. This leads the stabilized proteins to form aggresomes such as MDBs.

**Figure 5:**
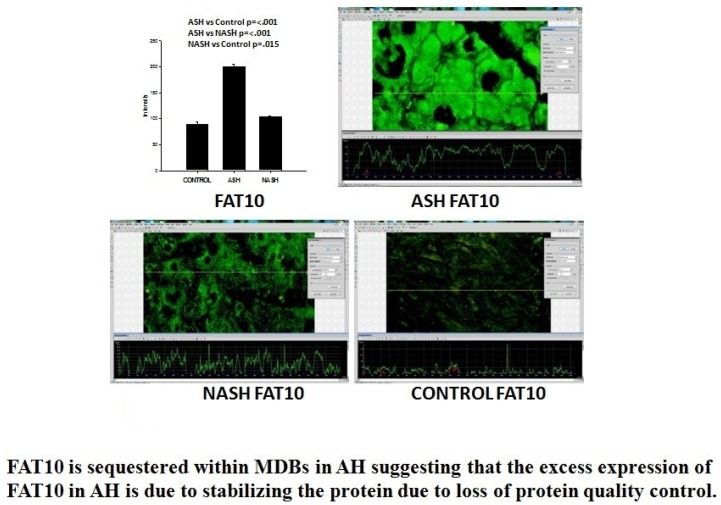
Graphs of FAT 10 fluorescent intensity staining measurements in Control, ASH and NASH. The intensity in ASH vs. control is *p* ≤ 001; ASH vs. NASH *p* ≤ 001; NASH vs. control is *p* = 015. Immunohistochemistry slides FAT10 in human biopsies ASH, NASH and Control. FAT10 is sequestered within MDBs in AH, suggesting that the excess expression of FAT10 in AH is due to the stabilizing of the protein due to a loss of protein quality control.

The FAT10 KO mice fed DDC did not develop the decrease in the 26S proteasome, whereas the wild type DDC refed did. Consequently, digestion of polyubiquinated proteins to form aggresomes (MDBs) did not occur in the FAT10 KO mice. Chymotrypsin was diminished in the wild type DDC. In the other groups, it was not diminished. This indicates that FAT10 WD mice fed DDC were prevented from forming a decrease in the 26S proteasome catalytic subunit because of the absence of the FAT10 dependent decrease in the expression of Beta5 (Figure 5).

The pathway that increases FAT10 expression includes TNFα and IFNγ, followed by NFκB and STAT3, all of which were up-regulated in alcoholic hepatitis. FAT10 over-expression in AH causes an extensive change in protein content in the liver, leading to balloon degeneration and MDB aggregation formation, all of which is prevented in KOFAT mice [45]. 

The patients with AH studied here were in a clinical trial for treatment where liver biopsies were done (Table 1), and the biopsies were used to quantitate the proteins by measuring the amount of protein by fluorescent intensity staining in order to determine the total protein, including the FAT10 stabilized protein. Using this technology, we can measure the amount of each protein in the control livers, in the AH livers, and the NASH livers simultaneously for comparison.

The immunofluorescence staining shows the FAT10 protein where the liver cell cytoplasm and nucleus of the hepatocytes are filled with FAT10. The expression of FAT10 was 4.5-fold increased in the AH livers, but not in the NASH or control livers. Other proteins in the Fatylation pathway (Utcl, Ufml, Uba5, Uba6 were significantly decreased [48].

The increase in ubiquitinated protein is demonstrated using Western blot. This reduction of proteolysis by the 26S proteasome, by the absence of KOFAT10 in mice, is demonstrated by a diminished rate of proteolysis in the lives of mice fed DDC compared to the control wild type mice 26S proteasome chymotrypsin activity [45]. This stabilization of proteins adds to the length of time of turnover and the apparent overexpression of proteins like CYP2E1, HNE, and GSH, which leads to oxidative stress when alcohol is consumed.

There are many gene over-expression pathways of FAT10, which are up-regulated by NFκB activation. Integren, lypo-poly-sacharidae (LPS), and SYK genes signal through TEC to NFκB and STAT3. The FAT10 gene is over-expressed partly because of a feedback loop where both have a NFκB gene present where it stimulates the FAT10 expression [49]. Immunofluorescent antibody staining of the AH livers shows localization of SYK almost exclusively in the cytoplasm of the balloon cell hepatocytes which have formed MDBs, suggesting that SYK remains where it was expressed by the stabilization of the protein. SYK is a very active protein with 10 phosphorylation sites. It plays a role in immune cell signaling pathways and liver fibrosis [50].

## 5. The Role of Alcohol-Induced Microtubule Hyperacetylation in ASH Progression

Alcohol metabolism by CYP2E1 produces the highly-reactive metabolite acetaldehyde, but also leads to extensive ROS and the ultimate production of other highly reactive metabolites. In the end, these metabolites form covalent modifications on DNA, lipids, and proteins, which can lead to modified functions of these macromolecules. Thus, we and others have postulated that the accumulation of these adducts in numbers and with time can lead to serious hepatic dysfunction and contribute to the progression of liver disease. More recently, alcohol exposure has been shown to induce post-translational modifications that are part of the natural repertoire, including methylation, phosphorylation and acetylation [51,52,53,54,55,56,57,58,59]. In particular, numerous proteins are known to be hyperacetylated on lysine residues upon ethanol exposure, and this list is expanding rapidly [59]. Using a proteomics approach, we identified 40 non-nuclear proteins that are hyper-acetylated in livers from ethanol-fed rats [55,60]. The reversibility of lysine acetylation and its presence on an ever-expanding list of proteins coupled with the numerous de-acetylases and acetyl-transferases that catalyze the reaction suggests that acetylation may rival phosphorylation in its ability to regulate cellular processes [61]. Thus, alcohol-induced protein acetylation likely leads to major physiological consequences that contribute to hepatotoxicity. 

Over the last two decades, we have been investigating the consequences of alcohol-induced microtubule acetylation on proper hepatic function. This line of investigation is based on our finding that microtubules display enhanced acetylation and are more stable in polarized hepatic WIF-B cells when chronically exposed to physiological concentrations of ethanol. We further determined that liver slices and livers from ethanol-fed rodents displayed a similar phenotype [55]. We have gone on to provide evidence that this alcohol-induced microtubule acetylation can explain known defects in key microtubule-based protein trafficking steps, including basolateral secretion and resident protein delivery [62], apically-directed transcytosis of newly-synthesized proteins [63] and the nuclear translocation of the STAT family of transcription factors [64]. We have also provided evidence that these trafficking defects likely result from impaired microtubule motor interactions with microtubules that impair vesicle motility along the polymer [63]. More recently, we have turned our attention to how alcohol-induced acetylation of cortactin may specifically impair the early steps of clathrin-mediated endocytosis by preventing dynamin recruitment to the necks of nascent clathrin-coated vesicles as they are budding from the basolateral surface [60,65]. 

A fatty liver (steatosis) is an early stage of alcoholic liver disease that is characterized by the formation and accumulation of enlarged hepatic lipid droplets. Although droplets are known to enhance liver injury, little is known about the mechanistic basis for this toxicity. Several recent lines of evidence indicate that microtubules are critical for regulating the lipid droplet life cycle at nearly every step, from their budding from the ER to their degradation by autophagy or droplet dispersal and lipolysis [66,67]. Droplets are also highly motile structures, and their bidirectional motility requires intact microtubules and is mediated by the microtubule-based motors, dynein or kinesin [68,69].

Because tubulin has been identified in the proteomes of purified droplets from dozens of sources including liver [70], it is considered a core droplet component [66,71]. Thus, we propose that alcohol-induced microtubule acetylation leads to altered lipid droplet dynamics, thereby contributing to enhanced steatosis. To test this hypothesis, we examined droplet dynamics in polarized hepatic cells treated with oleic acid (OA), a hepatotoxic fatty acid associated with the “Western” diet, in the presence or absence of ethanol. For our studies, we used WIF-B cells as an excellent cell culture model system for the investigation of alcohol-induced hepatotoxicity [72,73]. WIF-B cells fully polarize and differentiate after a week in culture. They form canalicular and sinusoidal membrane domains and function as adult hepatocytes, including the ability to metabolize alcohol using endogenously-expressed CYP2E1, ADH and ALDH [72,73]. As shown by others [74], we determined that lipid droplets were not only significantly larger in cells treated with OA and ethanol, but they were also far more numerous. Additionally, we determined that these large lipid droplets persisted upon ethanol withdrawal, suggesting impaired droplet degradation. Using a pharmacological approach, we determined that ADH-mediated (but not CYP2E1-mediated) ethanol metabolism was required for enhanced droplet accumulation. Live cell imaging of BODIPY-labeled droplets further revealed that droplet motility was virtually eliminated in cells treated with ethanol, except for a minority (~20% of total) of peri-nuclear distributed small droplets. Droplets in cells treated with nocodazole (a microtubule depolymerizing agent) were also stationary in both control and ethanol-treated cells, confirming microtubule-based motility. Additionally, the dynein and dynactin colocalized with the large, immotile droplets in ethanol/OA-treated cells.

To directly assess the relationship between microtubule acetylation and defects in lipid droplet motility, we exogenously expressed α-TAT1, the microtubule specific acetylase, using recombinant adenovirus. In cells over-expressing levels of STAT1 that led to enhanced microtubule acetylation as seen in ethanol-treated cells, we determined that lipid droplet dynamics were reduced in cells over-expressing STAT1, but to a lesser extent than that observed in ethanol-treated cells. As observed for ethanol-treated cells, dynactin/dynein colocalized with the immotile and enlarged lipid droplets, suggesting that its droplet-binding properties were impaired. Together, these results demonstrate that microtubule acetylation alone can explain defects in microtubule motor-dependent lipid dynamics. Because ethanol-treated cells displayed greater motility defects and because tubulin is known to be readily adducted by acetaldehyde [75], we propose that tubulin is additionally modified by reactive alcohol metabolites, leading to impaired motility. Together, these studies suggest that modulating the cellular acetylation levels with drugs currently in clinical trials for treating other metabolic diseases [76,77,78,79] could provide novel therapeutic strategies for treating alcoholic liver disease.

## 6. Micro-RNA Profiling of Hepatic Macrophages in Alcoholic Liver Disease

Alcoholic liver disease develops in approximately 20% of all alcoholics, with a higher prevalence in females [80]. There is a growing appreciation that innate immunity and inter-organ cross-talk contribute to ethanol-induced liver injury. Interactions between the intestine and liver are of particular importance. Alcohol exposure, in both people and rodents, impairs the barrier function of the intestine; this contributes to the progression of ALD [81]. Impaired gut integrity, in combination with dysregulation of the intestinal microbiome, leads to increased inflammatory responses in the liver. The hepatic macrophage, or Kupffer cell, is a critical mediator of hepatic inflammation in response to alcohol. Toll-like receptor 4 (TLR4) is activated by LPS coming from the gut; this response is exacerbated after ethanol exposure, as the Kupffer cells exhibit increased sensitivity to L LPS. 

During development, milk provides important factors that can promote growth and organ and immune system development [81]. Milk consists of carbohydrates, proteins, and fatty acids, and these all work together to protect the infant from pathogens and develop a healthy microbiota [82,83]. Human milk oligosaccharides (HMOs), including hyaluronan (HA), are beneficial glycans that promote healthy commensal bacteria and pathogen protection within the infant gut [81,84,85]. 

HA is an abundant extracellular matrix component of vertebrates, and is produced as a straight chain polymer strictly composed of repeating disaccharides of d-glucuronic acid and *N*-acetylglucosamine. HA is formed at cell surfaces by one or more hyaluronan synthases (HAS1, HAS2 and HAS3) embedded in their plasma membranes [85,86]. HA communicates with many cell types in a size-specific manner, using at least four signaling receptors including:

(1) CD44, a ubiquitously-expressed cell surface protein that recognizes HA sizes greater than 8 sugar moieties in length [87];
(2) RHAMM (receptor for HA mediated motility), which is important in signaling cell migration [88];
(3) Toll-like receptor (TLR) pattern recognition molecules TLR4 and TLR2 [89,90].

HA acts to recruit and activate leukocytes under pathological inflammatory settings and HA is now included among the damage-associated molecular pattern molecules (DAMPs) recognized in innate immunity [91,92]. Despite the potent ability of HA to stimulate inflammatory responses [91,92] specific-sized HA fragments can also have protective, anti-inflammatory effects [88]. Indeed, we have recently reported that HA fragments of an average molecular weight of 35 kD (HA35) normalizes TLR4-mediated signaling in Kupffer cells after chronic ethanol exposure [93]. Interestingly, HA35 is the specific-sized HA fragment that also drives epithelial bacterial defense mechanisms in the intestine [88].

There is a growing appreciation that miRNAs are critical regulators of TLR4 signaling [94]. Therefore, we used Next Generation Sequencing of miRNAs in Kupffer cells to identify miRNAs whose expression was reciprocally regulated by ethanol and HA35. Ethanol feeding down-regulated 30 miRNAs by more than two-fold; of these miRNAs, HA35 treatment restored the expression of 3 miRNAs [93]. Gene targeting analysis identified all 3 of these miRNAs restored by HA35 to be involved in the nuclear-cytoplasmic shuttling pathway, with miR-362 regulating expression of KPNA4 (importin α3), miR181b-3p regulating KPNA1 (importin α5) and miR-34* regulatingTNPO1 (transportin 1). 

We further investigated the interactions between miR181b-3p and importin α5 [94]. Kupffer cells expressed more importin α5 protein after ethanol feeding and HA35 treatment or overexpression of miR181b-3p prevented this response and also normalized LPS-stimulated TNFα expression in Kupffer cells from ethanol-fed rats. When mice were fed ethanol as part of their diet, hepatic miR181b-3p was decreased and importin α5 expression was increased in the immune cells of the liver.

These studies identified a miR181b-3p-importin α5 axis regulated in response to ethanol and HA35, revealing that changes in nuclear-cytoplasmic shuttling in Kupffer cells impacts the regulation of inflammatory signals.

## 7. Signature Pathway of Alcoholic Hepatitis—Integrative Global Analysis 

Alcoholic hepatitis is a unique spectrum of ALD with high mortality, for which effective therapy is currently limited to liver transplantation [95]. To identify novel therapeutic targets for AH, global “omic” analyses have been utilized for patient biopsy specimens or animal model tissues [96,97].

In applying such approaches, it is critical to search for differentially-regulated transcripts or proteins comparing AH to the ALD pathology, not to healthy livers. This way, what drives a shift from the precursor pathology to neutrophilic hepatitis will be scrutinized. Unfortunately, there is paucity in clinical data as to what constitutes this precursor lesion as it usually is asymptomatic and is not commonly presented to clinics and diagnosed. Nonetheless, this precursor lesion is believed to be chronic alcoholic steatohepatitis (ASH) often accompanying liver fibrosis or cirrhosis. We were able to reproduce in mice chronic ASH characterized by macro- and micro-vesicular steatosis, balloon-cell degeneration, mononuclear cell inflammation, pericellular and perisinusoidal liver fibrosis [98]. By adding the weekly binge, this chronic pathology shifted to AH with neutrophilic infiltration, thus providing a model which allows global search for AH drivers. 

Examination of gene expression profiles of AH vs. chronic ASH livers shows predicted upregulation of genes expressed by neutrophils, as well as chemokines responsible for neutrophilic infiltration including osteopontin (*Spp1*). SPP1 was previously implicated in neutrophilic inflammation in the NIAAA binge model. In this previous study, *Spp1* knockout mice were protected from neutrophilic infiltration [99]. 

In contrast, SPP1 deficiency in our model not only aggravated hepatitis in some mice but also converted chronic hepatitis to AH without weekly binge [97], highlighting the pleiotropic nature of SPP1 expressed by multiple cell types. 

Another pathway evident in the AH versus chronic ASH model is the so-called “ductular reaction”. This is histologically-detectable by a cluster of cells forming the ductal lumens, which presumably reflects the proliferation of terminal ductular cells and correlates with a poor prognosis of AH patients [100]. 

Genes known to be markers of these cells, *Epcam*, *Sox9*, *Ck19*, etc, are highly unregulated in AH livers as compared to chronic ASH. These cells are also regarded as oval progenitor cells as they are positive for the A6 antigen and appear without an obvious ductal formation. Both the function or origin of these progenitor cells are presently elusive. Genes involved in extracellular vesicles (EVs) are upregulated in AH vs. chronic ASH livers, suggesting the release of EVs is heightened in the former livers. As EVs are already shown to serve as a pivotal mechanism of pro-inflammatory or pro-fibrogenic actions, the roles of EVs in AH, particularly neutrophilic infiltration and fibrogenesis, are expected.

Genes known to respond to infection are also clustered as upregulated genes in AH. In particular, *Casp11* (*CASP4* in man) is induced in the pathway categories of both infection and inflammation. This is because this caspase becomes activated by intracellular pathogens or PAMPs, cleaves and activates Gasdermin-D (GSDMD), and causes programmed lytic cell death called pyroptosis. As this mode of cell death results in the release of bacteria and PAMPs into the local environment, intense neutrophilic inflammation is likely induced. 

Gut dysbiosis, impaired intestinal mucosal barrier, and bacterial translocation are known pathogenetic events of alcoholic liver disease [101]. The CASP11-GSDM-D pathway may be a consequence of these events and a cause of neutrophilic infiltration in AH.

## 8. Gut-Liver Challenge in Alcoholic Liver Disease

Chronic alcohol consumption is associated with quantitative changes in intestinal bacteria. Intestinal overgrowth is present in the small and large intestine of mice fed alcohol intragastrically for 3 weeks as compared with isocaloric fed mice. This was assessed using culture-dependent and -independent methods. Compositional changes in the intestinal microbiome are characterized by the suppression of several beneficial bacteria, including *Lactobacillus,* as assessed by deep sequencing of a conserved region in the bacterial 16S ribosomal RNA (rRNA) gene [102]. Similar findings were observed in alcohol-dependent patients. Dysbiotic changes were partially reversible after 3 weeks of alcohol abstinence in the fecal microbiota [103]. 

Bacterial colonization of mucosal surfaces in the duodenum was increased in patients with alcohol abuse as compared with non-alcoholic controls as assessed by quantitative PCR (qPCR), suggesting that small intestinal overgrowth is not only restricted to the luminal compartment, but also occurs with adherent bacteria [101]. 

The intestinal microbiota contains bacteria, fungi and viruses. Chronic alcohol abuse induced intestinal fungal dysbiosis in humans. Healthy subjects had a high diversity of fungi in the feces as assessed by using internal transcribed spacer (ITS) sequencing. Fungal diversity was decreased and overgrowth of *Candida* was observed in alcohol dependent patients with mild disease, patients with alcoholic hepatitis or cirrhosis. Compared with non-alcoholic controls and patients with non–alcohol-related cirrhosis, alcoholic cirrhotic individuals showed an increased systemic exposure and immune response to mycobiota as measured by serum anti–*Saccharomyces cerevisiae* IgG antibodies (ASCA) [104].

How do changes in the intestinal microbiome contribute to ALD? Dysbiosis-induced intestinal inflammation contributes to a disruption of intestinal tight junctions and onset of increased intestinal permeability, which allows microbial products to translocate to the liver, induce inflammation and cause a progression of liver disease. Metagenomic and metabolomic changes in the intestine contribute to a gut barrier dysfunction as well. Saturated long-chain fatty acids and short-chain fatty acids are reduced in intestinal contents of ethanol-fed rodents. Supplementation of these bacterial metabolites stabilizes the gut barrier and reduces alcohol-induced liver disease [105,106]. 

How do changes in the gut microbiota occur? Use of gastric acid-suppressing medications such as proton-pump inhibitors (PPI) is increasing worldwide, as is the incidence of chronic liver disease. Evidence from mice and humans suggests that gastric acid suppression alters specific bacteria in the intestinal microbiota to promote liver injury and progression of ethanol-induced liver disease (chronic Lieber DeCarli feeding model), fatty liver disease (high-fat diet feeding), and non-alcoholic steatohepatitis (choline-deficient diet feeding). This study used *Atp4a^Sl/Sl^* mice, which have a point mutation in the gastric H+, K+-ATP-ase α subunit that leads to achlorhydria, as well as mice given PPIs. Mice with gastric acid suppression developed alterations to the intestinal microbiota—specifically increases in *Enterococcus* species—that promoted hepatic inflammation and liver injury. Increased progression of ethanol-induced liver disease, non-alcoholic liver disease and steatohepatitis was observed in these mice. *Enterococcus* was found to act directly on Kupffer cells and hepatocytes in vitro. Furthermore, in a cohort of 4830 chronic alcohol abusers, active users of PPIs had a statistically significant higher rate of liver disease than the previous users or never-users. Thus, gastric acid suppression is causing intestinal dysbiosis, which is associated with the progression of chronic liver disease [107].

## 9. Liver Influences on Alcohol Drinking Behavior

The interactions between the liver and brain, with a major focus on inflammatory disease and the immune system, e.g., chronic inflammatory diseases, can be associated with behavioral alterations [108]. Both peripherally-released cytokines and peripheral monocytes have been suggested to have the potential to modify brain function, in part by activating brain microglia. Neuroinflammation has also recently been a particular focus with respect to alcohol actions and alcohol consumption, and several studies have suggested a role for cytokines and chemokines as modulators of alcohol drinking [109]. 

We have used an unbiased systems genetic approach to identify liver secretion products that can influence the predisposition for voluntary alcohol consumption. Our approach is based on the use of a panel of recombinant inbred (RI) rat strains, the HXB/BXH RI panel [110]. RI panels are a valuable resource for systems genetic research. These panels comprise rat (or mouse) strains that are isogenic within the strain, while each strain is a different genetic mosaic of its parental strains. Because the strains are inbred, phenotypic data can be accumulated over time on rats with the same genotype, and the genetic relationship among the strains provides an opportunity to detect and map genetically-mediated influences on these phenotypes. 

We previously measured voluntary alcohol consumption by rats from the HXB/BXH RI panel [111], and we have generated whole-genome transcriptome data for brain, liver and other organs of naïve (no ethanol exposure) rats from the RI panel (data available at http://phenogen.ucdenver.edu). The transcriptome data were analyzed to identify gene coexpression networks using Weighted Gene Coexpression Network Analysis (WGCNA) [110,112]. These coexpression networks reflect the relatedness among expression levels of various transcripts, and the networks are organized into “modules” of transcripts with expression levels that are highly correlated across the rat strains. It has been established that the correlation of transcript expression levels across different environments (genetic backgrounds of the rats in the RI panel) indicates that these correlated transcripts are likely involved in similar biological processes, i.e., are functionally related.

The genetic influence on a phenotype can occur due to DNA polymorphisms that produce a variation in protein coding regions, leading to altered protein function, or, more commonly, as a result of polymorphisms in regulatory regions, leading to changes in RNA expression levels. In many cases, differences in gene expression levels have been found to underlie differences in complex phenotypic traits. Given our phenotype data on alcohol consumption by rats in the RI panel, and the transcriptional networks/coexpression modules that we have identified in various organs and tissues of the alcohol naïve rats (http://Phenogen.ucdenver.edu), http://phenogen.ucdenver.edu to http://phenogen.org, one is in a position to “mine” our transcriptional data to determine if quantitative differences in the transcriptome, particularly as deduced from coexpression modules, are associated with predisposition to various levels of alcohol consumption.

Our criteria for identification of a predisposing transcriptional module are: 1) the expression levels of transcripts in the module (summarized by the first principal component, the module “eigenegene”) are significantly correlated with the phenotype across the HXB/BXH RI panel; 2) the module eigengene has a significant quantitative trait locus (QTL, a region of the genome that regulates the quantitative trait of transcript expression levels); and 3) the module eigengene QTL overlaps a QTL for the correlated phenotype, i.e., the same region of the genome that regulates alcohol consumption also regulates the expression levels of the transcripts in the correlated co-expression module.

This requirement arises from the premise that if the transcripts in the co-expression module contribute to alcohol consumption through their levels of expression, then the genetic factors/regions that regulate their expression levels should be the same as the genetic factors that regulate alcohol consumption levels.

Since expression levels of transcripts do differ in various organs or tissues, investigators generally choose to investigate transcript levels in organs that are “appropriate” in relation to the phenotype being studied. In the case of voluntary alcohol consumption, the usual choice has been the brain, and we have previously published data on a brain transcriptional module whose eigengene QTL overlapped the QTL for alcohol consumption [110]. However, the QTL analysis of alcohol consumption behavior resulted in two QTL “peaks”, one on chromosome 1 and the other on chromosome 12. The brain co-expression module explained the behavioral QTL on chromosome 12, but the behavioral QTL on chromosome 1 did not overlap with the eigengene QTL of any brain module. Given the evidence for liver-brain communication, we “mined” our liver transcriptome data and surprisingly identified a liver module that met all the criteria for being a contributor to the predisposition for escalating alcohol consumption.

The transcripts in the liver module associated with alcohol drinking are functionally related, and code for liver secretory products that influence appetite, lipid metabolism and inflammation. The levels of these secretory products in the naïve rats can predispose the animals to different levels of alcohol consumption.

Our studies illustrate how data mining of genetically-regulated transcriptional networks, coupled with a systematic, holistic approach to the identification of genetically-influenced behavioral phenotypes, can lead to the discovery of novel genetically controlled systems that predispose complex behaviors, even through organs that may not *a priori* appear to be candidates for affecting the behavior.

## 10. Phenotyping Human CYP1A1 Polymorphisms Using Model Organisms

CYP1A1 and CYP1A2 polymorphisms were expressed in budding yeast (*Saccharomyces cerevisiae*) to determine whether particular polymorphisms that are linked to human disease confer higher levels of carcinogen-associated genotoxicity compared to the common or “wild type” allele. The carcinogens studied included the hepatocarcinogen aflatoxin B1 (AFB1), the lung carcinogen benzo(a)pyrene 8, 7 dihydrodiol (BaP-DHD), and the colon-associated carcinogen 2-amino-3-methyl-imidazo (4,5-*f*) quinilone (IQ). The CYP polymorphisms that we studied included CYP1A1 I462V and CYP1A1 T461N, as well as CYPIA2 D348N, CYP1A2 I386F, and CYP1A2 C406Y; the CYP1A1 polymorphisms were studied because their incidence is linked to lung, breast, and endometrial cancer [113]. Genotoxicity endpoints included DNA repair foci, DNA adduct formation, growth inhibition, and recombination [114]. We observed that the CYP1A1 polymorphism, CYP1A1 I461V that was linked to a higher incidence of human lung cancer, actually conferred lower levels of genotoxicity in yeast exposed to carcinogens, compared to CYP1A1. These data are in agreement with studies involving CYP1A1 knock-out mice, indicating that CYP1A1 is protective and that other P450 enzymes, such as CYP1B1, may encode the P450 enzyme that activates lung cancer-associated carcinogens.

Additional studies were performed to phenotype rare CYP1A2 polymorphisms and we showed that, in agreement with studies performed in *Escherichia coli*, CYP1A2 I386F polymorphism conferred a lower level of genotoxicity. These studies indicate that phenotypic properties of CYP alleles can be characterized in budding yeast and provide additional evidence that CYP1A1 may be protective.

CYP450 enzymes are involved in the metabolism of multiple drugs, hormones, and xenobiotics. The genes that encode these enzymes are highly polymorphic and their expression is also dependent on lifestyle factors, such as smoking, diet, and alcohol consumption.

A salient question has been whether particular P450 polymorphisms are correlated to diseases, such as cancer and alcoholism, in which there is strong evidence that risk factors include exposure to environmental carcinogens and diet. For example, CYP2E1 activity has been known to aggravate alcohol toxicity, and some studies have suggested that high activity alleles of CYP2E1 may be more common in alcoholics. Particular CYP1A1 alleles have been associated with lung cancer, whose incidence is highly correlated to environmental exposure of agents, such as polyaromatic hydrocarbons (PAHs). However, many alleles are very rare, rendering it difficult to ascribe associations using epidemiological studies alone.

One current hypothesis is that CYP alleles associated with lung cancer confer higher levels of catalytic activity that convert PAHs into highly-active genotoxins. Testing this hypothesis using mammalian cells that express these CYP alleles has been complicated by expression of many additional phase I and phase II enzymes that inactivate genotoxins. Indeed, mouse knockouts of CYP1A1 are actually less susceptible to PAHs, suggesting that phase II enzymes may coordinate with CYP1A1 to rapidly detoxify xenobiotic compounds. This has led to the hypothesis that CYP1A1 may be protective.

To elucidate these questions, we expressed CYP1A1 alleles individually in budding yeast, which do not contain CYPs that can activate the carcinogens. Due to short generation times and well-described genetics, budding yeast is useful for measuring multiple genotoxic endpoints. However, due to differences in gene regulation mechanisms, we studied alleles that only map in the amino acid coding sequence.

We used site-specific mutagenesis of CYP1A1 and CYP1A2 to generate CYP1A1 462V, CYP1A1 T461N, CYPIA2 D348N, CYP1A2 I386F, and CYP1A2 C406Y in multi-copied expression plasmids. All the alleles were confirmed by the DNA sequences. These CYP expression plasmids were then introduced into yeast strains by transformation. After exposure to the carcinogen or solvent control, we measured genotoxic endpoints, including recombination, DNA repair foci formation, DNA adduct formation, and growth inhibition. Growth inhibition was measured in a double mutant, *rad4 rad51*, defective in both nucleotide excision repair and recombinational repair, which is extremely sensitive to DNA damage; A_600_ was measured in real-time over a period of 24 h using a Tecan plate reader. Recombination was measured in yeast strains that contained two *his3* fragments, *his3- Δ3′* and *his3- Δ5′*; recombination between these fragments generates His^+^ recombinants; the spontaneous frequencies are ~10^-7^, rendering it possible to detect small increases in recombination frequencies. DNA adduct formation was measured by isolating DNA from yeast cells exposed to the carcinogen, hydrolyzing the DNA, and performing mass spectroscopy analysis. Rad51 foci formation was measured in strains that expressed yellow-fluorescent protein-tagged (yfp)-Rad51. Foci were visualized and counted from images obtained in a confocal microscope [115,116].

Our studies were focused on two sets of CYP polymorphisms; CYP1A1 polymorphisms I462V and T461N, and CYP1A2 polymorphisms, D348N, I386F, and C406Y. We introduced plasmids containing these polymorphisms into yeast strains to measure genotoxic endpoints.

Regarding CYP1A1 alleles, we observed that expression of CYP1A1, CYP1A1 I462V or CYP1A1 T461N can activate carcinogens and significantly inhibit the growth of the DNA repair mutant *rad4 rad51* and stimulate carcinogen-associated recombination frequencies. However, growth inhibition was greater for CYP1A1 compared to either CYP1A1 I462V or CYP1A1 T461N after exposure to either AFB1 or BaP-DHD [116].

**Figure 6:**
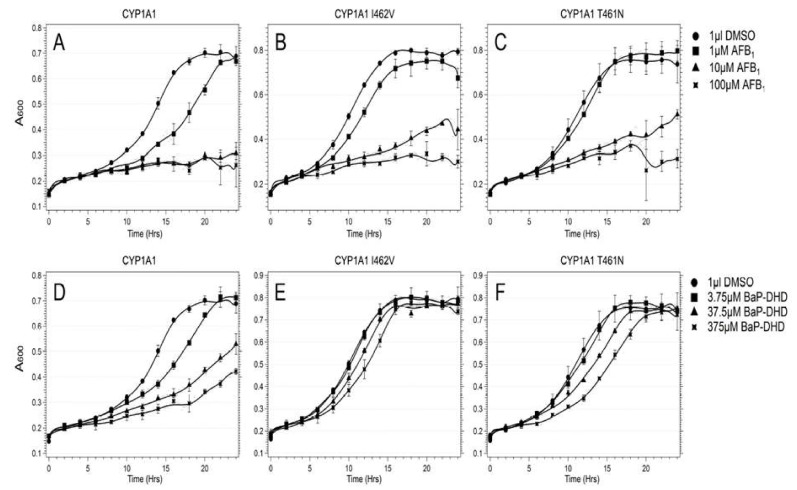
Expression of CYP1A1 alleles in budding yeast activates BaP-DHD and AFB1 to become potent genotoxins. Growth of the *rad4 rad51* strain expressing CYP1A1 (**A**,**D**), CYP1A1 I462V (**B**,**E**), CYP1A1 T461N (**C**,**F**) was measured in real-time after exposure to either AFB1 (top) or BaP-DHD (bottom). A_600_ is plotted against time (hrs).

In addition, carcinogen associated recombination frequencies and Rad51 foci formation were lower in cells expressing CYP1A2 I462V, compared to cells expressing CYP1A1. As controls for these studies, we determined ethoxy-resorufin deethylase (EROD) activities in cells expressing all these CYP1A1 variants and showed that all these P450 alleles still confer robust levels of activity. A representation of this data is shown in Figure 6 [116].

Regarding CYP1A2 alleles, we observed that expression of CYP1A2 I386F conferred little genotoxic activity compared to CYP1A2, CYP1A2 D348N or CYP1A2 C406Y. This was evidenced by the low levels of AFB1-associated DNA adducts, AFB1-associated recombination frequencies, and recombination foci in AFB1-exposed cells compared to cells expressing the other CYP1A2 alleles. However, we did not observe any clear statistical difference in numbers of genotoxic endpoints in comparing carcinogen-exposed cells expressing CYP1A2, CYP1A2 C406Y, or CYPA2 D348N. Controls for these experiments included detecting all the CYP1A2 variants using Western blots and also measuring methyl-resorufin demethylase (MROD) activity in cells expressing CYP1A2, C406Y, and D348N variants. The budding yeast is useful in phenotyping human P450 polymorphisms, as demonstrated by multiple genotoxic endpoints can be readily measured. These studies have elucidated observations made in mouse CYP1A1 knockouts, which exhibit lower frequencies of carcinogen-associated tumors.

We suggest that CYPIA1 I462V and T461N may be associated with higher incidences of cancer by exhibiting lower activity or affinity to the xenobiotic compounds. Other P450 enzymes, which are not coupled with phase II activities to detoxify these compounds, may then activate the compounds to potent genotoxins [116].

## 11. Alcohol and Non-Infectious Liver Diseases

There is a diversity of alcohol-induced liver damage. However, since the alcohol is absorbed by the gastrointestinal system, the health of the gastric and intestinal mucosa will determine the blood alcohol levels. Moreover, intestinal bacterial overgrowth may lead to sugar fermentation, resulting in measurable blood alcohol levels [14,117].

The toxicity of alcohol targets several organs, with the most damage being done to the liver. It is there that toxic metabolites of ethanol act upon hepatocytes and non-parenchymal cells, as well as intrahepatic granulocytes, lymphocytes, and monocytes [117,118,119,120,121]. Experimental models, as well as human studies, exist which analyze the various stages of the central nervous system, behavior, gastro-intestinal damage and genetics [122,123,124,125]. Alcohol consumption is increasing the severity of infectious diseases [124]. In addition, chronic alcohol consumption is involved in rare diseases [125]. There is a need to reduce the rising burden of injury due to alcohol, obesity, and drugs of misuse. Since alcohol is metabolized differently by diverse ethnic populations, it is important to personalize the therapeutic strategy [126]. The most recent systematic review and meta-analysis demonstrates that the risk for incidence of liver cirrhosis for former drinkers in comparison to long-term abstainers was three-fold higher [127]. Moreover, since the risk of liver cirrhosis increases exponentially among women, the care of women should represent a priority for precision medicine [127,128].

ALD is prominent in the iron overload diseases: thalassemia, hemochromatosis, *porphyria cutanea tarda* [128,129,130,131,132,133,134,135]. Patients with hemochromatosis (C282Y homozygosity) misuse alcohol, and a high proportion of patients with *porphyria cutanea tarda* are often also heavy drinkers [133,134,135].

Alcohol consumption in individuals with thalassemia has a synergistic effect with iron-induced liver damage [136]. We report here an episode of alcohol misuse in a 34-year-old individual with asymptomatic thalassemia. The next day after consuming alcohol, the man was hospitalized with clinical symptoms of liver damage. The patient’s anamnesis taken by a physician familiar with the disease revealed that he had first degree relatives diagnosed with beta-thalassemia. Upon a physical examination, he presented an enlarged liver. The assessment of serum concentration of alanine amino transferase (ALT) and determination of serum ferritin concentration were compatible with thalassemia. Molecular genetic testing in the pathogenic variants was prescribed.

## 12. Alcohol and Therapeutics Interaction

The use of medication, either prescribed or over the counter, may influence the P450 of the gut and activate or inhibit its actions.

Interactions between alcohol and prescription drugs are common, particularly the additive effects with benzodiazepines and also with some of the antihistamine drugs; other interactions may occur with tricyclic antidepressants [137]. An important additional element of the hepatotoxic threat of alcohol is its enhancement of the hepatotoxic effects of other hepatotoxins (e.g., CCl_4_) and even some drugs (e.g., acetaminophen) [138,139,140,141,142,143,144,145,146,147,148].

Highly active antiretroviral therapy (HAART) is used to treat human immunodeficiency virus (HIV) infection in HIV patients. Alcohol consumption can interact with HAART as well as other pharmaceutical agents used for the prevention of opportunistic infections [149].

## 13. Conclusions

Alcoholic-induced organ damage, as well as the MEOS, are subjects of continuous research and review of research [146,147]. It is mandatory that clinical, basic, and translational scientists collaborate on a common agenda for the future. The priorities for this collaboration should be to better understand the impact of alcohol and other drug-related organ injuries, for clinicians to accurately identify those products that cause injury, and for biochemists and toxicologists to isolate and test the products’ ingredients for their toxic potential. Ultimately, the findings from the collaborations must be used to notify regulators with information necessary to guide the development of safer products and the removal of injurious products from the market.

## Figures and Tables

**Table 1:** Patients with AH studied for changes in the expression of FAT10.

#	Fat Macro	Fat Micro	PMN	Lymph	Other Findings	Fibrosis Stage	MDBs
01-011	1+	0	0	0	0	4+	1+
01-015	3+	1+	1+	1+	0	4+	3+
01-016	4+	0	3+	1+	bile thrombi, EM	4+	4+
03-001	1+	0	3+	4+	bile thrombi, EM	4+	4+
03-005	2+	0	0	4+	EM	4+	1+
03-006	1+	0	4+	1+	satellitosis, EM	4+	4+
03-007	1+	0	0	2+	0	4+	1+
03-012	2+	0	3+	3+	best satellitosis, EM	4+	4+
03-014	2+	0	2+	0	most MDBs	4+	4+
03-015	4+	0	1+	0	0	4+	2+
03-017	3+	1+	2+	3+	EM	3+	1+
03-018	4+	0	4+	2+	Satellitosis	4+	3+
03-019	3+	0	3+	0	satellitosis, EM	4+	4+
03-020	1+	0	3+	0	Satellitosis	4+	1+
03-022	3+	1+	0	2+	autophagy, EM	4+	3+
03-023	1+	0	4+ satellitosis	1+	autophagy of MDBs, EM	4+	4+
03-024	4+	1+	3+	1+	PMN lymphocytes, EM	4+	4+
03-025	4+	1+	1+	1+	autophagy, EM	3+	3+
03-027	1+	0	4+	4+	PMN satellitosis	4+	4+
03-039	0	0	1+	4+	duct lymphocytes PMN	4+	0

In all biopsies duct metaplasia was positive.

## Abbreviations

ABCA1	ATP-binding cassette transporter
ABCG1	ATP-binding cassette sub-family G member 1
ACC	Acetyl-CoA carboxylase
ADH	alcohol dehydrogenase
ADR	Adiponectin
AdipoR	Adiponectin receptor
AH	acute alcoholic hepatitis
ALD	alcoholic liver disease
ALDH	acetaldehyde dehydrogenase
ALF	alcoholic fatty liver
ALT	Alanine aminotransferase
AMPK	5′AMP-activated protein kinase
ApoCII	Apolipoprotein C2
ASH	alcoholic steato-hepatitis
α-SMA	Alpha-smooth muscle actin
AST	aspartate aminotransferase
ATM	ataxia-telangiectasis-mutated
ATR ataxia and rad	ataxia and rad-3 related
AUDIT	Alcohol Use Disorders Identification
CCl4	Carbon tetrachloride
CDT	carbohydrate-deficient transferrin
Chk	check point kinase
ChREBP	Carbohydrate response element-binding protein
Col	Collagen type 1
CPT	Carnitine palmitoyl-transferase
Ctsb	Capthepsin B
CYP	cytochrome P450
DAMPS	damage-associated molecular patterns
EMA	European Medicines Agency
FABP	Fatty acid-binding protein
FAS	Fatty acid synthase
FAT 10	human leukocyte antigen F locus adjacent transcript 10
FDA	Food and drug administration
FFA	Free fatty acid
Fibroscan	Vibration-Controlled Transient Elastography
FMT	Fecal microbial transplantation
FXR	farsenoid X receptor
G6Pase	Glucose 6-phosphate
GAPDH	Glyceraldehyde-3-phosphate dehydrogenase
GAA	guanidino-acetate
GAMT	guanidino-acetate methyltransferase
GGPP	Geranyl-geranyl pyrophosphate
γGT	gamma glutamyl transpeptidase, Gamma-glutamyl-transferase
GLUT4	Glucose transporter type 4
GNA15	canine nucleotide-binding protein Alpha 15
GPx	Glutathione peroxidase
GSDMD	Gasdermin-D
GSH	Glutathione
HBV	hepatitis B virus
HCC	hepatocellular carcinoma
HCV	hepatitis C virus
HDL	high density lipoprotein
HFD	high fat diet
HMOs	Human milk oligosaccharides
4-HNE	4-hydroxynonenal
IKKβ	Inhibitor of nuclear factor kappa-B kinase subunit beta
IARC	International Agency for Research on Cancer
IGF	insulin-like growth factor
IL	Interleukin
IL	Interleukin 8—CXCL8
iNOS	Nitric oxide synthase
ITS	internal transcribed spacer
LDL	Low-density lipoprotein
LA	linoleic acid
LCT	long-chain triglycerides
LDL	low density lipoprotein
LGG	*Lactobacillus rhamnosus* GG
LPS	Lipopolysaccharide
MAPK	Mitogen-activated protein kinases
MCD	Methionine-choline-deficient diet
MCP-1	Monocyte chemotactic protein 1
MCT	medium-chain triglycerides
MCV	mean corpuscular volume of erythrocytes
MD2	Myeloid differentiation 2
MDA	Malondialdehyde
MDB	Mallory-Denk body
MDF	Maddrey discriminator function
MELD	Model for End-Stage Liver Disease
MEOS	microsomal ethanol oxidizing system
MONCF	monocyte-derived neutrophil chemotactic factor
MRI	magnetic resonance imaging
NAFLD	Non-alcoholic fatty liver diseases
NAS	NALFD activity score
NASH	Non-alcoholic steatohepatitis
NEFA	Non-esterified fatty acid
NFκB	Nuclear factor kappa-B
OA	Oleic acid
PAMPS	Pathogen-associated molecular patterns
PCR	Polymerase chain reaction
PMN	Poly-morpho-neutrophyl
PPAR	Peroxisome proliferator-activated receptor
PPI	proton pump inhibitor
ROS	reactive oxygen species
SAH	S-adenosyl-homocysteine
SAM	S-adenosyl-methionine
SCD	Stearoyl-CoA desaturase
SF	saturated fat
SHP	Small heterodimer partner
SIBO	small intestinal bacterial overgrowth
SNAP	single nucleotide polymorphisms
SOD	Superoxide dismutase
SREBP1	Sterol regulatory element-binding protein 1
TBARS	Thiobarbituric acid reactive substances
TC	Total cholesterol
TG	Total triglycerides
TGF-β	Transforming growth factor beta
TIMP	Tissue inhibitor of metalloproteinase
TLR	Toll like receptor
TNF	Tumor necrosis factor
UPN	Upper the normal values
USF	unsaturated fat
WGCNA	Weighted Gene Coexpression Network Analysis
